# An atypical presentation of Wilms tumor as duodenal obstruction in a toddler: a case report and review of literature

**DOI:** 10.1093/jscr/rjag656

**Published:** 2026-07-29

**Authors:** Deekshya Devkota, Amrita Pandey, Suman Bikram Adhikari, Bal Mukunda Basnet, Shree Krishna Poudel

**Affiliations:** Department of Pediatric Surgery, Kanti Children’s Hospital, Kathmandu, Nepal; Arghakhanchi District Hospital, Arghakhanchi, Nepal; Department of Pediatric Surgery, Kanti Children’s Hospital, Kathmandu, Nepal; Department of Pediatric Surgery, Kanti Children’s Hospital, Kathmandu, Nepal; Nepalese Army Institute of Health Sciences, Kathmandu, Nepal

**Keywords:** case report, duodenal obstruction, radical nephrectomy, Roux-en-Y gastrojejunostomy, toddler, Wilms tumor

## Abstract

Wilms tumor is the most common primary renal malignancy in children, typically presenting as an asymptomatic abdominal mass in children between 2 and 6 years of age. Duodenal obstruction secondary to direct tumor compression is an exceptionally rare presentation and poses a diagnostic and surgical challenge. We report a 2.5-year-old male who presented with a 1-week history of abdominal pain, abdominal distension, and bilious vomiting. Imaging revealed a large right-sided Wilms tumor causing compression at the duodenojejunal junction. The patient underwent emergency exploratory laparotomy with right radical nephrectomy and Roux-en-Y gastrojejunostomy. Histopathology confirmed Stage III Wilms tumor with favorable histology. The patient recovered well and tolerated subsequent adjuvant chemotherapy and radiotherapy. This case highlights that proximal intestinal obstruction may rarely be the first manifestation of Wilms tumor. Although neoadjuvant chemotherapy before surgical excision constitutes the standard treatment approach at our institution, upfront surgical resection was undertaken to relieve the duodenal obstruction.

## Introduction

Wilms tumor is the most common primary malignant renal tumor of childhood. It typically presents between 2 and 6 years of age as an asymptomatic abdominal mass, sometimes accompanied by abdominal pain, anemia, hematuria, hypertension, or nonspecific gastrointestinal symptoms [1, 2]. Intestinal obstruction is a recognized postoperative complication following nephrectomy for Wilms tumor, with reported incidences around 5%–7% [3]. In contrast, presentation with proximal bowel obstruction as a direct consequence of the primary tumor’s mass effect is strikingly rare. Only isolated neonatal and early-infant cases have described Wilms tumor compressing the small bowel loops to produce features of intestinal obstruction, usually managed with nephrectomy [4, 5].

Here we present an atypical case of a 2.5-year-old male with primary duodenal obstruction and subserosal bleeding caused by the mass effect of a right Wilms tumor without metastasis, managed by exploratory laparotomy with right radical nephrectomy and gastrojejunostomy. This case report adheres to the SCARE 2025 guidelines [6] and presents the patient’s clinical presentation, investigations, and management.

## Case presentation

A 2.5-year-old male presented to the emergency department with a 1-week history of abdominal pain, progressive abdominal distension, and bilious vomiting. Abdominal pain was acute in onset, generalized, dull aching in nature, and associated with multiple episodes of non-projectile, bilious vomiting. On presentation, blood pressure was elevated at 120/80 mmHg; all other vital parameters were within normal limits. On abdominal examination, the abdomen was soft, non-tender, and mildly distended. A well-defined, firm mass, with a smooth surface measuring approximately 6 × 4 cm, was palpable in the right lumbar region of the abdomen which was ballotable and moved along with respiration. Laboratory investigations at admission revealed moderate anemia, reactive thrombocytosis, transaminitis with hypoalbuminemia, and elevated inflammatory markers (Table 1).

**Table 1:** The patient’s laboratory workup at admission

Laboratory parameters	Values	Reference range
WBC	7760/cumm	4000–11 000/cumm
Neutrophils	74%	40%–75%
Hemoglobin	8.4 g/dl	11.5-18 g/dl
Platelets	565 000/cumm	150 000–400 000/cumm
CRP	17.7 mg/L	<5 mg/L
TB/DB	0.5/0.36 mg/dl	0.3–1.2/0–0.4 mg/dl
ALT	94 U/L	0–41 U/L
ALP	59 U/L	82–350 U/L
AST	136 U/L	0–45 U/L
Ur/Cr	77/0.68 mg/dl	13–45/0.3–1.1 mg/dl
Na/K	153/3.97 mg/dl	135–145/3.5–5.5 mg/dl
PT	13 s	9.0–13.0 s
INR	1.18	0.9–1.3
APTT	38.4 s	24–41 s
Serum calcium	7.82 mg/dl	8–10.4 mg/dl
Corrected calcium for albumin	9.5 mg/dl	
Serum phosphorus	4.01 mg/dl	4–7 mg/dl
Serum uric acid	5.4 mg/dl	1.5–7 mg/dl
Serum albumin	1.88 g/dl	3.5–5.5 g/dl
Total protein	3.89 g/dl	5.5–8.5 g/dl
LDH	442 mg/dl	135–400 mg/dl
Blood grouping	A positive	
Serology	Serology	

WBC, white blood cells; Hb, hemoglobin; PLT, platelets; PT/INR, prothrombin time/international normalized ratio; APTT, activated partial thromboplastin time; TB/DB, total bilirubin/direct bilirubin; ALT, alkaline aminotransferase; AST, aspartate aminotransferase; ALP, alkaline phosphatase; LDH, lactate dehydrogenase; CRP, C-reactive protein.

**Figure 1:**
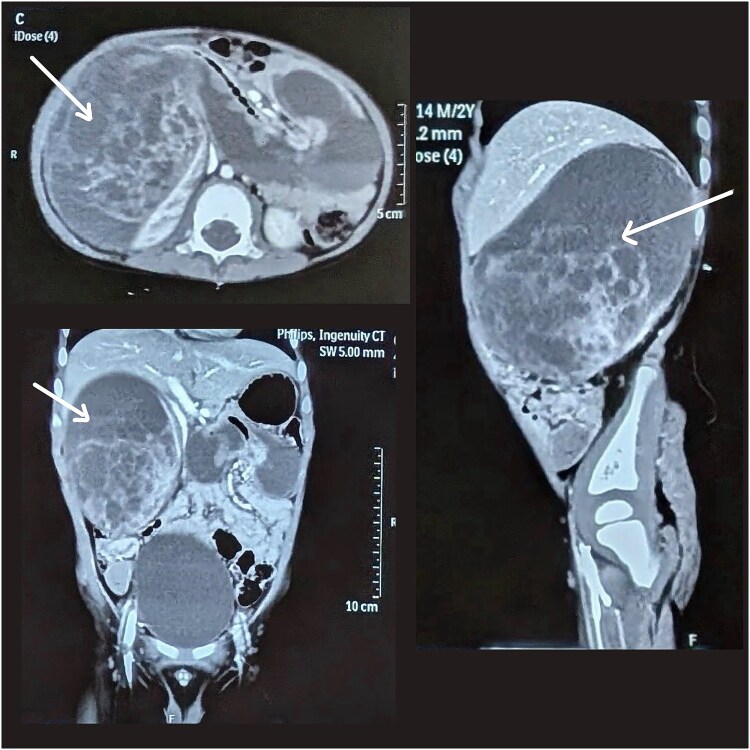
Axial, coronal, and sagittal sections of a CT scan showing a large heterogeneously hypodense space-occupying lesion in the right kidney, suggestive of Wilm tumor of the right kidney.

Contrast-enhanced computed tomography (CT) of the abdomen and pelvis demonstrated a large heterogeneously hypodense lesion measuring 11 × 8.5 × 12.2 cm in the right kidney, displacing the duodenum superolaterally (Fig. 1). The features were consistent with Wilms tumor of the right kidney without metastasis. The imaging also revealed duodenal stenosis at the duodenojejunal junction, associated with marked dilatation of the third and fourth parts of the duodenum, consistent with proximal intestinal obstruction.

The patient was managed with intravenous antibiotics, analgesics, and antihypertensive therapy (Tab Amlodipine 1.2 mg) for persistent hypertension. A multidisciplinary evaluation involving the pediatric oncology, pediatric ICU, and cardiology team was undertaken.

At our institution, the standard treatment protocol for Wilms tumor involves neoadjuvant chemotherapy followed by surgical resection as per the International Society of Paediatric Oncology (SIOP) approach. However, given the clinical and radiological evidence of duodenal obstruction in this case, a multidisciplinary discussion led to the decision for emergency surgical intervention.

**Figure 2:**
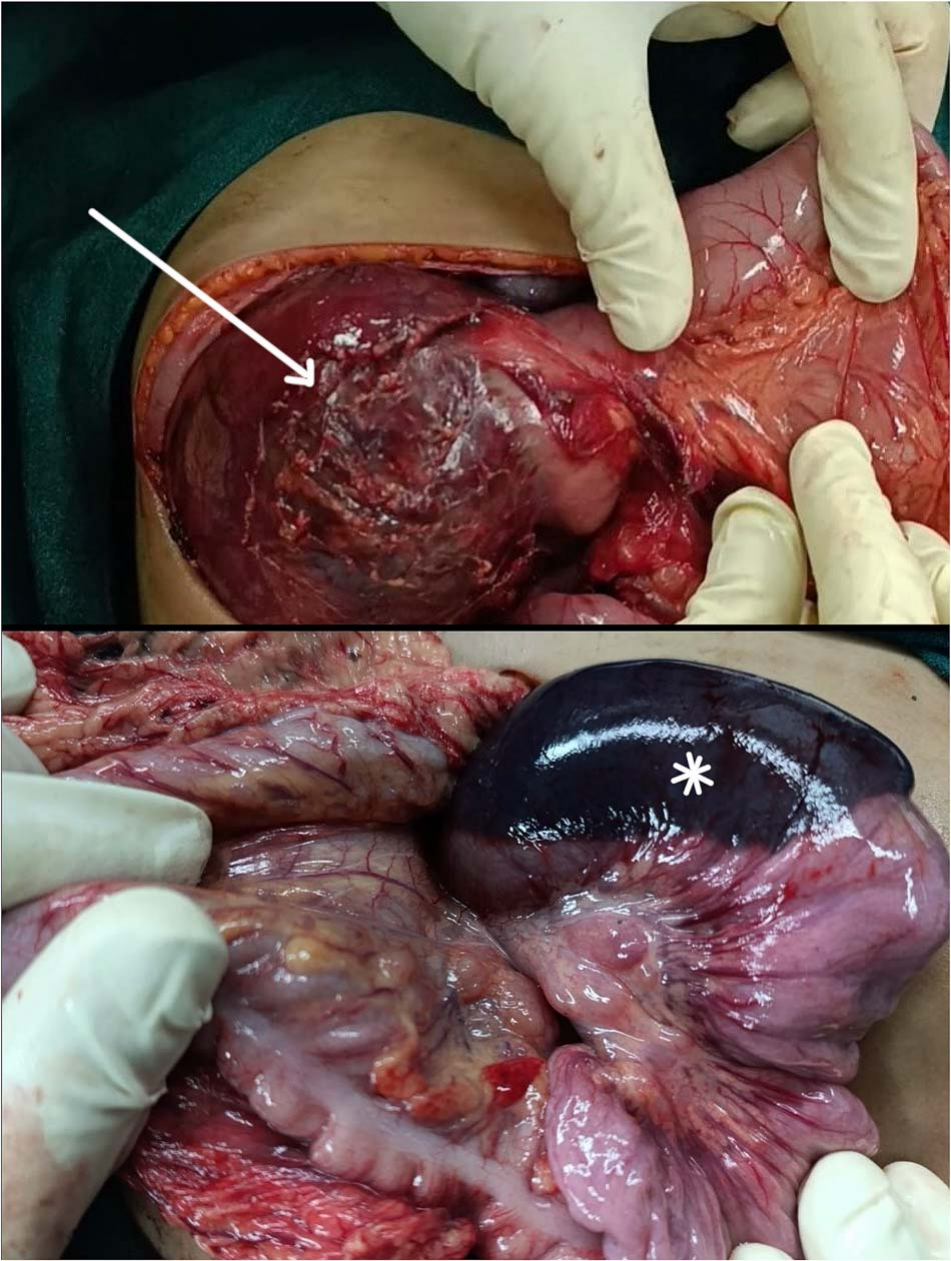
A highly vascular mass in the right retroperitoneum arising from the right kidney with complete obliteration of the renal parenchyma and hematoma formation within (arrow). The mass was adherent to the second part of the duodenum, resulting in subserosal hemorrhage (asterisk).

The patient underwent emergency exploratory laparotomy, with right radical nephrectomy and Roux-en-Y gastrojejunostomy to relieve the duodenal obstruction. Intraoperative findings include a highly vascular mass of around 10 × 7 × 6 cm in the right retroperitoneum arising from the right kidney with complete obliteration of the renal parenchyma with hematoma formation within (Fig. 2).

The mass was firmly adherent to the second part of the duodenum, with visible subserosal hemorrhage at the duodenal wall caused by extrinsic compression from the tumor. The duodenal wall itself was intact and there was no full-thickness involvement, perforation, or intrinsic duodenal pathology so the duodenum was not resected. Enterotomy was performed to exclude concomitant duodenal atresia (Type I), which was not identified.

Given the dense adhesion of the tumor to the periduodenal tissues and the resulting duodenal obstruction at the duodenojejunal junction, primary duodenoduodenostomy or duodenojejunostomy was not feasible. A Roux-en-Y gastrojejunostomy was therefore created to provide a reliable, tension-free bypass of the obstructed segment: the Roux configuration was selected specifically to prevent bile reflux gastritis. Right radical nephrectomy was then completed with the tumor mass en bloc. There was minimal intraoperative tumor spillage. The mass had formed a pseudocapsule with the right adrenal gland; the adrenal gland was included in the resection specimen.

Histopathological examination revealed features consistent with Wilms tumor with favorable histology. Renal sinus involvement and capsular invasion were present; however, there was no evidence of lymphovascular invasion or anaplasia. Based on the clinical, radiological, and histopathological details, this case was consistent with Stage III Wilms tumor.

**Figure 3:**
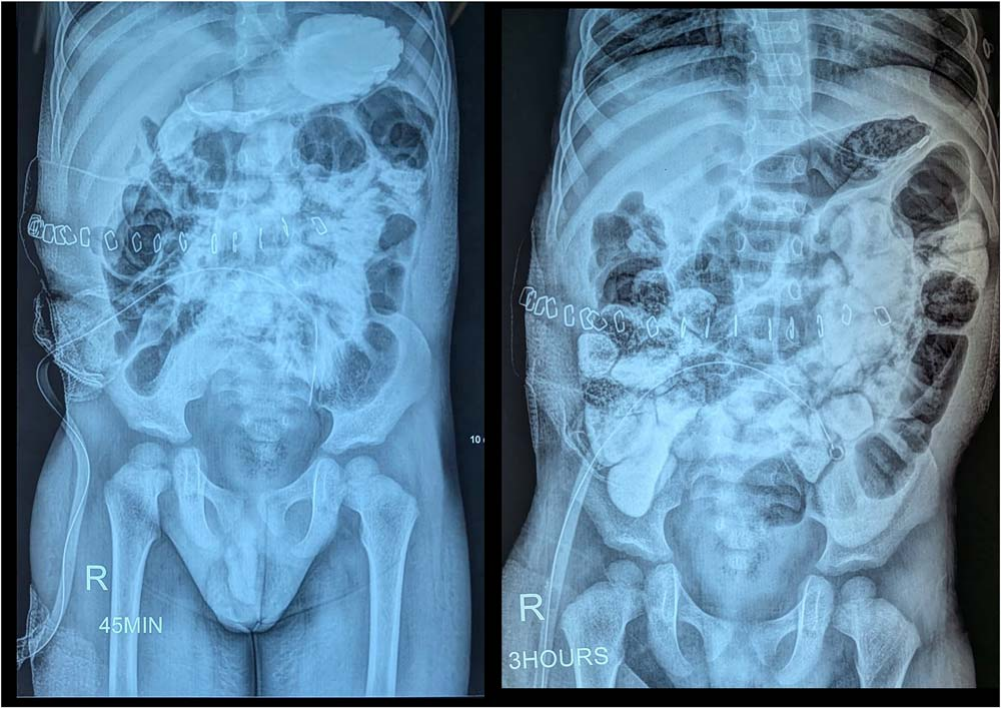
Contrast-enhanced abdominal radiographs at 45 min and 3 h showing gradual passage of contrast through the small bowel to the colon, suggesting resolution of obstruction.

A repeat contrast follow-through performed on the 7th post-operative day (POD) demonstrated gradual passage of contrast into the colon, consistent with resolution of the small bowel obstruction (Fig. 3). The patient demonstrated satisfactory clinical recovery with stable hemodynamic parameters and adequate urine output. Chemotherapy with Injection Vincristine was started on the 12th POD, and the patient tolerated it well. At the three-month follow-up, the patient completed 4th cycle of chemotherapy and 14 days of flank radiotherapy. On the 14th day of radiotherapy, the patient developed acute radiation enteritis, which was managed conservatively.

## Discussion

The classic presentation of Wilms tumor is a painless abdominal mass, which may be accompanied by abdominal pain, hematuria, and hypertension [7]. In our case, the patient presented with acute-onset abdominal pain, progressive abdominal distension, and bilious vomiting. A right-sided Wilms tumor was identified, exerting mass effect on the duodenojejunal junction and producing features consistent with proximal bowel obstruction. While bowel obstruction is a recognized postoperative complication following nephrectomy, its occurrence as the initial presenting manifestation of Wilms tumor is unusual [3].

Ahmed *et al.* [5] described a similar atypical presentation in a newborn girl who developed abdominal distension and recurrent meconium-stained vomiting on the second day of life. Intraoperatively, a cystic-solid retroperitoneal mass arising from the inferior pole of the left kidney was found to be displacing the small bowel and transverse colon. Similarly, Sandler *et al.* [8] reported a 5-year-old female with a 1-week history of abdominal pain, non-blood-stained, non-bilious vomiting, and hematuria, in whom imaging revealed a right-sided Wilms tumor associated with a completely obstructing intraluminal duodenojejunal hematoma.

Neonatal cases have described massive renal tumors displacing and compressing small bowel loops to produce upper gastrointestinal obstruction, relieved after nephrectomy alone [4, 5]. Our patient shared several features with these reports, a large unilateral renal mass, radiologic displacement and compression of proximal small bowel, and favorable histology. However, the presence of subserosal hemorrhage due to extrinsic compression is not well documented in prior reports.

Atypical presentations of Wilms tumor extend beyond gastrointestinal involvement. A 16-year-old female presenting with dyspnea, left shoulder tip pain, and unintentional weight loss was found on biopsy of a left chest mass to have metastatic Wilms tumor [9]. Large cohort studies and surgical guidelines focus on the management of bowel obstruction as a postoperative complication rather than a preoperative or primary presentation [10, 11].

At our institution, the management of Wilms tumor follows the SIOP approach, which advocates for a neoadjuvant chemotherapy protocol before nephrectomy, as preoperative chemotherapy leads to tumor size reduction, formation of a fibrous pseudocapsule facilitating surgical removal, and a decreased risk of intraoperative rupture and hemorrhage [12]. However, the patient developed acute duodenal obstruction, a surgical emergency that necessitated immediate surgery to relieve the obstruction, so neoadjuvant chemotherapy was not a feasible option.

The surgical management in our case, which combines oncologic resection with gastrointestinal bypass for obstruction, highlights the importance of individualized, multidisciplinary care in atypical presentations. Lack of long-term follow-up remains a major limitation of this case report, as the oncologic outcomes could not be assessed.

## Conclusion

This case illustrates an exceptionally rare presentation of Wilms tumor as duodenal obstruction in a toddler, rather than as the more typical asymptomatic abdominal mass or as postoperative obstruction. When acute surgical emergencies arise, adherence to the standard neoadjuvant chemotherapy protocols is not a feasible option, and upfront surgery becomes a necessary approach.

## Highlights

- Duodenal obstruction as the primary presenting manifestation of Wilms tumor is a rare occurrence and to our knowledge, this is among the first documented cases in a toddler beyond the neonatal period.
- Intraoperative findings revealed subserosal hemorrhage at the duodenal wall from direct tumor compression—a feature not previously described in prior reports of Wilms tumor associated bowel obstruction.
- Combined oncologic resection (right radical nephrectomy) with gastrointestinal bypass (Roux-en-Y gastrojejunostomy) was performed in a single-stage emergency setting, demonstrating the feasibility of simultaneous tumor excision and obstruction relief.
- This case highlights that acute surgical emergencies in Wilms tumor may necessitate deviation from standard neoadjuvant chemotherapy protocols, with upfront surgery serving as a lifesaving intervention.
